# Oxidative Stress in the Pathogenesis of Antiphospholipid Syndrome: Implications for the Atherothrombotic Process

**DOI:** 10.3390/antiox10111790

**Published:** 2021-11-09

**Authors:** Cristina Nocella, Simona Bartimoccia, Vittoria Cammisotto, Alessandra D’Amico, Daniele Pastori, Giacomo Frati, Sebastiano Sciarretta, Paolo Rosa, Chiara Felici, Oliviero Riggio, Antonella Calogero, Roberto Carnevale, SMiLe Group

**Affiliations:** 1 Department of Clinical Internal, Anesthesiological and Cardiovascular Sciences, Sapienza University of Rome, 00161 Rome, Italy; cristina.nocella@uniroma1.it (C.N.); daniele.pastori@uniroma1.it (D.P.); 2 Department of Medical-Surgical Sciences and Biotechnologies, Sapienza University of Rome, 04100 Latina, Italy; simona.bartimoccia@uniroma1.it (S.B.); fraticello@inwind.it (G.F.); sebastiano.sciarretta@uniroma1.it (S.S.); p.rosa@uniroma1.it (P.R.); chiara.feli@gmail.com (C.F.); antonella.calogero@uniroma1.it (A.C.); 3 Department of General Surgery and Surgical Specialty Paride Stefanini, Sapienza University of Rome, 00161 Rome, Italy; vittoria.cammisotto@uniroma1.it; 4 Department of Movement, Human and Health Sciences, University of Rome “Foro Italico”, 00135 Rome, Italy; a.damico@studenti.uniroma4.it; 5 Department of AngioCardioNeurology, IRCCS Neuromed, 86077 Pozzilli, Italy; 6 Department of Translational and Precision Medicine, “Sapienza” University of Rome, 00161 Rome, Italy; oliviero.riggio@uniroma1.it; 7 Faculty of Medicine and Surgery, Course E, Sapienza University of Rome, 04100 Latina, Italy; smilegrouplatina@libero.it; 8 Mediterranea, Cardiocentro, 80122 Napoli, Italy

**Keywords:** oxidative stress, thrombosis, antiphospholipid syndrome, antioxidant treatment

## Abstract

Atherothrombosis is a frequent complication of the clinical history of patients with antiphospholipid syndrome (APS). Both atherothrombosis and APS are characterized by increased oxidative stress. Oxidative modifications are implicated in the formation of antiphospholipid antibodies, which in turn may favour the oxidative imbalance by increasing the production of reactive oxidant species (ROS) or by a direct interaction with pro-oxidant/antioxidant enzymes. As a result of these processes, APS patients suffer from an oxidative imbalance that may contribute to the progression of the atherosclerotic process and to the onset of ischemic thrombotic complications. The aim of this review is to describe mechanisms implicated in the formation of ROS in APS patients and their involvement in the atherothrombotic process. We also provide an overview of potential therapeutic approaches to blunt oxidative stress and to prevent atherothrombotic complications in these patients.

## 1. Introduction

In the early 1980s, the term antiphospholipid syndrome (APS) was coined to describe an autoimmune, multisystemic disorder characterized clinically by autoantibody-induced thrombophilia [1]. Today, APS is considered an autoimmune, thrombo-inflammatory disease characterized by vascular thrombosis in the setting of one or more antiphospholipid antibodies (aPLs) such as lupus anticoagulant (AL), anticardiolipin antibodies (aCL) and anti-β2-glycoprotein1 antibodies (aβ2GPI) [2]. Beyond thrombosis, APS regularly manifests with other morbid features including thrombocytopenia, cardiac dysfunction [3], accelerated atherosclerosis, nephropathy, movement disorders, and cognitive decline [4,5]. This heterogeneous clinical presentation reflects the complex pathogenesis of APS, reinforcing the need for a deeper knowledge of mechanisms of aPL formation and of thrombotic complications, to allow a better-tailored, integrated, multidisciplinary approach.

It is known that the pathogenesis of APS consists of two phases: “the first hit and second hit”. According to this theory, the “first hit” is represented by the presence of circulating aPL that destroy the integrity of the endothelium inducing a procoagulant phenotype. Nevertheless, aPL alone are not enough to cause thrombosis, which takes place only in the presence of a triggering factors (the “second hit”), which is usually represented by smoking, acute infections, oxidative stress or inflammation [6].

Growing evidence from cellular, animal, and human studies provides the direct role of oxidative stress in atherothrombosis. Therefore, oxidative stress, as a second hit, could have a fundamental role in the progression of the APS.

In this review we will examine the contribution of oxidative stress in the pathogenesis of APS and in particular in the setting of atherothrombosis. Specifically, we will describe (1) the role of oxidative stress in atherothrombosis development, (2) clinical and experimental evidence of increased oxidative stress in these patients and (3) antioxidant supplementation as a potential treatment.

## 2. Mechanisms of Atherothrombosis in APS: The Role of Oxidative Stress

The diagnosis of APS requires the concomitant presence of vascular thrombosis and/or pregnancy morbidity [7], in addition to persistent positivity to at least one of the aPL among LA, aCL and aβ2GPI. However, some patients may present noncriteria antibodies or unusual clinical manifestations [8,9]. Venous thromboembolism is the most common clinical presentation of the syndrome whereas arterial thrombosis is less frequent and mainly affects younger adults. The clinical spectrum of arterial thrombosis may extend from asymptomatic small ischemic lesions to fully ischemic stroke [10]. An observational study in 1000 APS patients from 13 European countries, that were followed prospectively for 10 years, showed that thrombotic events appeared in 16.6% during the first 5 years and 14.4% during the second 5 years. The most common events reported were strokes, transient ischaemic attacks, deep vein thrombosis and pulmonary embolism [11].

It’s now well established that oxidative stress plays a major role in atherogenesis [12,13,14]. Oxidative stress is defined by an imbalance between reactive oxygen species (ROS) production and impaired detoxification by antioxidant enzymatic and nonenzymatic systems [15,16,17]. This imbalance characterizes several cardiovascular diseases (CVD) in which ROS are important mediators of endothelial damage leading to vascular inflammation and progression of the atherosclerotic plaque. The causal role of ROS in atherosclerosis and other cardiovascular diseases is supported by several animal models of oxidative stress.

**Figure 1:**
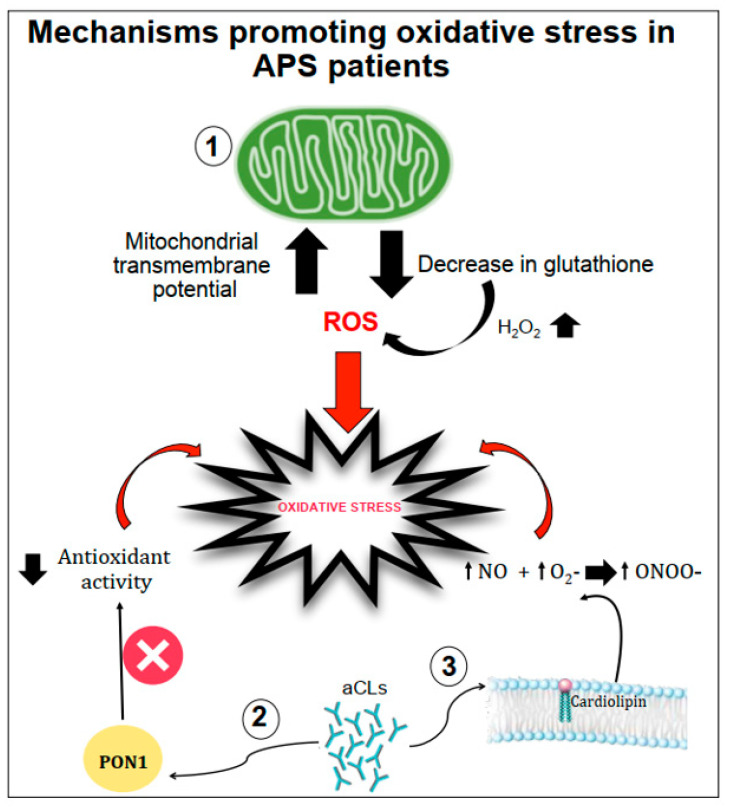
Schematic representation of mechanisms promoting oxidative stress in APS patients. Oxidative stress can be favoured by (1) the increase in the mitochondrial transmembrane potential and the decrease in intracellular glutathione contents; (2) the interactions between anticardiolipin antibodies (aCL) and the paraoxonase-1 (PON1) limiting its antioxidant properties; (3) aCL induction of nitric oxide (NO) and superoxide (O_2_^−^) production with increased levels of peroxynitrite (ONOO^−^) a pro-oxidant molecule.

Several mechanisms have been proposed as promoters of oxidative stress in APS patients (Figure 1).

Gergely et al. [18] verified, in patients with systemic lupus erythematosus, the hypothesis that the mitochondrial transmembrane potential and production of reactive oxygen intermediates (ROIs) mediate the imbalance of apoptosis which may significantly contribute to inflammation. In these patients, they found that mitochondrial transmembrane potential and ROI production were elevated compared to healthy subjects. Moreover, intracellular glutathione contents were diminished, and H_2_O_2_, a precursor of ROIs, increased mitochondrial transmembrane potential and caused apoptosis [18].

Another mechanism of oxidative stress can be related to the interactions between aCL antibodies and antioxidant enzymes in plasma, such as the paraoxonase-1 (PON1), which is an antioxidant enzyme linked to HDLs that prevents LDL oxidation. Indeed, in patients positive for aCL antibodies, the activity of PON1 was found to be dramatically decreased [19]. Charakida et al. [20] also confirmed the interactions between aCL antibodies and PON1. They showed that women with positive aPL antibodies have functional and structural arterial abnormalities that were associated with reduced activity of PON1. This implicates HDL and oxidative stress in the causal pathway for atherosclerosis in these patients. Moreover, in these patients, HDL has a “proatherogenic” phenotype by reducing nitric oxide bioavailability and impairing anti-inflammatory and antioxidant properties [20].

Finally, aCL seems to play an important role in promoting oxidative stress by inducing nitric oxide (NO) and superoxide production. This reaction favours enhanced production of plasma peroxynitrite, which is a powerful pro-oxidant substance. Indeed, in mice injected with aCL antibodies, there was an increase in serum nitrotyrosine suggesting that permanent pro-oxidant environment induces the activation of iNOS and results in long-term downregulation of iNOS expression and subsequent endothelial dysfunction [21].

**Figure 2:**
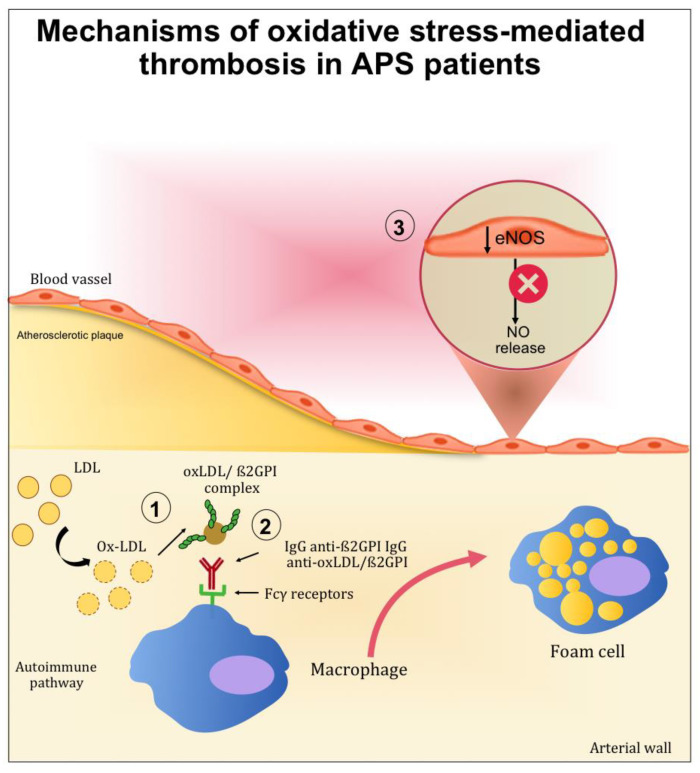
Mechanisms mediated by oxidative stress contributing to thrombotic complication in APS patients. (1) After oxidative modification, oxLDL binds β2GPI inside the arterial wall and further increases inflammation, oxidation, and cell activation. (2) Autoantibodies to this complex are produced resulting in circulating complexes (oxLDL/β2GPI/antibody). In the presence of anti-oxLDL/β2GPI antibodies, the uptake of oxLDL/β2GPI complexes by macrophage is increased and may further accelerate the development of atherosclerosis. (3) The endothelial nitric oxide synthase (eNOS) in the endothelial cells is inactivated, reducing the nitric oxide (NO) bioavailability.

When oxidative stress is established, it contributes significantly to the pathophysiology of APS by (1) inducing protein structural modification, and (2) interfering with nitric oxide metabolism (Figure 2).

### 2.1. The Role of Oxidative-Mediated Modifications

Clinical and epidemiological studies suggested that the presence of anti-β2GPI antibodies confers a significant risk of thrombosis, morbidity and mortality in young adults [22]. For this reason, anti-β2GPI antibodies have been widely investigated to better understand the pathophysiology of APS and its complications.

β2GPI is a 50 kDa protein synthesized as a single polypeptide chain. It is mainly produced in the liver and may be detected in the blood at a concentration of 200 µg/mL. [23]. β2GPI has a role in coagulation, fibrinolysis, angiogenesis, and apoptosis [24]. Oxidation and nitrosylation of redox-sensitive cysteine residues are characteristic post-translational modifications of β2GPI occurring under conditions of increased oxidative or nitrosative stress. In particular, modifications of the sulfhydryl group (SH) alter the function of proteins containing cysteines in their catalytic domain or as interface residues of interacting proteins. ROS readily react with cysteine residues, especially redox-active cysteines, to form reversible or irreversible oxidized forms.

S-nitrosylation refers to a chemical reaction that occurs spontaneously or enzymatically in the presence of high NO concentrations. As a covalent post-translational modification on the cysteine thiol residue, s-nitrosylation has emerged as an important mechanism for functional regulation of most or all main classes of protein and intracellular processes [25]. These post-translational modifications directly affect the function of β2GPI and also confer an increase in the immunogenicity of β2GPI. In particular, the oxidation of β2GPI may increase the immunogenicity of the molecule by (1) increasing the affinity of anti-β2GPI antibodies to oxidised β2GPI; (2) causing immature monocyte-derived dendritic cells to mature that secret interleukin (IL)-12, IL-1, IL-6, IL-8, tumour necrosis factor-α and IL-10; (3) breaking immune tolerance [26].

The contribution of ROS to the development of APS has been studied in the context of lipid peroxidation and the formation of oxidized LDL (oxLDL)/β2GPI complexes. Indeed, patients with systemic autoimmune diseases displayed increased lipid peroxidation and oxLDL production [27,28]. After oxidative modification, electrostatic forces initially mediate the bind between oxLDL and β2GPI. After this initial interaction, more stable complexes (non-dissociable) are formed and stabilized by covalent interactions. These complexes are both proatherogenic and immunogenic. Indeed, the binding of β2GPI to oxLDL may occur inside the intima microenvironment of the arterial wall and further increase inflammation, oxidation, cell activation and macrophage uptake of oxLDL/β2GPI complexes [29]. Moreover, patients with SLE and APS produce autoantibodies to this complex [30], and the resulting circulating immune complexes (oxLDL/β2GPI/antibody) may further accelerate the development of atherosclerosis. This was demonstrated in vitro by the increased uptake of oxLDL/β2GPI complexes by macrophage in the presence of anti-oxLDL/β2GPI antibodies [31,32]. These results provide an explanation for the accelerated development of atherosclerosis in autoimmune patients.

### 2.2. Role of Oxidative Stress in Nitric Oxide Metabolism

Among the mechanisms potentially implicated in oxidative stress-mediated atherothrombotic complications in APS, the inactivation of endothelial nitric oxide synthase (eNOS) is one of the most studied. eNOS is the predominant NOS isoform in the vasculature and is responsible for most of the nitric oxide (NO) produced in this tissue. NO is a short-lived gas molecule that is responsible for different biological actions in multiple tissues and cell types, and is synthesized by eNOS to preserve vascular homeostasis [33]. Moreover, it exerts an atheroprotective function, and it inhibits blood clots and platelets adhesion to the endothelium [34].

A hypothetical connection between APS and alterations in the NO bioavailability has been evaluated in several studies performed both in mice and humans [35,36,37,38]. The evidence resulting from these studies showed a direct connection between the altered production of NO and APS pathogenesis.

In patients with aPL, a negative correlation was found between urinary NO metabolites (NOx) and IgG anticardiolipin, suggesting that aPL can negatively affect NO physiological activities [39].

In mice, the injections of polyclonal aPL and β2GPI monoclonal antibodies isolated from human patients can reduce the plasma concentration of NO metabolites. Moreover, the injection of aPL suppressed eNOS-mediated vascular relaxation by acetylcholine. Finally, in mice lacking eNOS, the increase in leukocyte adhesion to vascular endothelium and thrombus formation induced by aPL was not observed [36].

In vitro studies carried out by Ramesh et al. showed the impact of aPL in endothelial cells. In mice, aPL produced an increase of monocyte adhesion to endothelial cells, which is a mechanism directly related to atherosclerosis [36]. The same authors examined the role of β2GPI in aPL antagonism to eNOS by experiments that alternately included and excluded β2GPI from the surface of endothelial cells. When these cells were deprived of β2GPI, aPL did not cause eNOS inhibition, indicating that β2GPI is required for aPL full functioning [36,37,38].

## 3. Oxidative Stress in APS: Clinical and Experimental Studies

Several clinical studies evaluated oxidative stress biomarkers in APS patients (Table 1).

aPLs have been demonstrated to induce an increased expression of molecules able to produce an expanded oxidative status in plasma, as demonstrated by high levels of prostaglandin F2-isoprostanes in APS patients. F2-isoprostanes are arachidonic acid products formed on membrane phospholipids by the action of ROS. As F2-isoprostanes are characterized by stability and specificity for lipid peroxidation, they represent a reliable marker for quantitative measurement of lipid peroxidation oxidative stress in vivo and prediction of cardiovascular events [53,54]. Specifically, a study conducted on 45 APS patients found higher values of 8-isoprostanes in the APS group than in the other groups. Moreover, APS patients with enhanced inflammation and oxidative stress recorded more thrombotic events compared to control group (69% vs. 6.5%). Similar results were showed by another study that investigates the relationship between oxidative stress and monocyte tissue factor (TF) expression in a cross-sectional comparison of aPL-positive and aPL-negative patients [41]. In fact, in these patients, an upregulation of monocyte TF expression was associated with thrombosis [55]. The results showed that compared with aPL-negative subjects, in aPL-positive patients higher values of isoprostanes and monocyte TF antigen and activity were observed [41].

PON1 is a hydrolytic enzyme with wide range of substrates, and a capability to protect against lipid oxidation. There is a considerable in vitro and in vivo data that prove the beneficial effects of PON1 in several atherosclerosis-related processes [56].

In an observational study, among 56 patients with APS, 37 presented arterial thrombosis, 16 presented venous thrombosis and all showed malondialdehyde-modified LDL (MDA-LDL) at significantly higher levels than controls. Furthermore, basal serum of PON1 activity was dramatically decreased in a subgroup of patients in comparison with the controls [19]. These results suggest that PON1 abnormalities that play a role in the APS might be associated with a higher risk of arterial thrombosis. Genetic analysis confirmed the role of PON1 in APS. In fact, PON1 L55M polymorphism resulted in an association with APS [47].

Consistently with these results, a cross-sectional study showed that PON1 is reduced in 36 patients with primary APS compared with 20 healthy subjects (HS) [40]. Additionally, the total antioxidant capacity (TAC), which quantifies the overall antioxidant defence of plasma, analysed in patients with primary APS did not differ significantly from levels in the control group, but correlated positively with PON activity [40].

Chronic, autoimmune, vascular inflammation together with decreased PON activity may contribute to oxidative stress, LDL modification (oxLDL) and oxLDL/2GPI complex formation that reflect the oxidative stress degree. Matsuura et al. revealed that serum levels of IgG anti-oxLDL/2GPI antibodies were significantly higher in systemic lupus erythematosus (SLE) patients with APS compared to SLE controls without APS. In addition, high concentrations of these IgG antibodies were observed in APS patients with a history of arterial thrombosis. Therefore, the presence of circulating oxLDL/2GPI complexes and IgG antibodies to these complexes indicates significant vascular damage and oxidative stress as well as a significant role in autoimmune-mediated atherothrombosis [42].

A cross-sectional case–control clinical study, including a total of 180 patients with primary and secondary APS and a control group, investigated several oxidative stress markers of endothelial damage measured by flow-mediated dilation (FMD). Biomarkers of oxidative stress, lipid hydroperoxydes (LOOH), advanced oxidation protein products (AOPP), total sulfhydryl groups (tSHG), and PON1 activity resulted altered in APS patients [45].

In addition to the evidence from human models, some studies on murine models supported that enhanced oxidative stress occurs in APS and the role of oxidant/antioxidant balance.

Severe combined immunodeficiency (SCID) mice were injected with Hybridomas producing human and murine aCL antibodies and β2GPI monoclonal antibodies. Results showed that PON1 activity, NO levels, and expression of total antioxidant capacity (TAC) were reduced. Conversely, peroxynitrite and superoxide concentration in plasma were increased. These data confirm that aCL antibodies are associated with the decreased PON activity and reduced endothelial function that may occur in the APS [49].

At the molecular level, it has been demonstrated that in the liver of the APS mouse model, both mRNA and protein expression of 47phox, a protein involved in the upregulation of nicotinamide adenine dinucleotide phosphate (NADPH) oxidase activity, were increased compared with the control group [51]. As NADPH oxidase is the main source of ROS [57], the results suggest that NADPH oxidase-mediated oxidative stress leads to endothelial cell injury in APS.

The studies described so far analyse the oxidative stress at plasmatic levels. Perez-Sanchez et al. studied oxidative stress at the cellular level by analysing biomarkers in circulating leucocytes from APS patients. Higher peroxide production, the nuclear abundance of Nrf2, antioxidant enzymatic activity, decreased intracellular glutathione, and altered mitochondrial membrane potential were found in monocytes and neutrophils from APS patients compared to healthy subjects [58]. Specifically, ROS production was markedly increased in monocytes and neutrophils of APS patients compared with healthy donors, as was the expression of Nrf2, the main regulator of antioxidant genes. Moreover, intracellular reduced GSH was significantly decreased in both cell types and the activities of catalase (CAT) and glutathione peroxidase (GPx) resulted in being strongly reduced. Furthermore, a significant systemic reduction of total antioxidant capacity (TAC) in plasma from APS patients was found compared to healthy donors, and might indicate a reduced ability to counteract ROS production and oxidative damage [58].

According to the results of these studies, showing that oxidative stress is directly involved in the pathophysiology of atherothrombosis in APS, the evaluation of oxidative stress biomarkers could be used as serologic indicators to assess the APS patient’s risk for vascular complications. Moreover, vascular, preventive strategies and more targeted therapeutic interventions should be developed.

## 4. Antioxidant Treatment in APS Patients: The State of the Art

Common treatments for APS are long-term anticoagulation with vitamin K antagonists and antiplatelet drugs. To reinforce the effects of these therapies and to fight the effects of oxidative stress in APS, several potential new therapeutic approaches are under investigation. Strategies to inhibit oxidative stress involve drugs such as dabigatran and statins that, with different molecular mechanisms, can reduce vascular oxidative stress and inflammation and improve endothelial function.

A potential new therapeutic strategy should be represented by natural molecules such as vitamins, CoQ10, and omega-3 polyunsaturated fatty acid (n-3 PUFA) (Table 2).

### 4.1. Human Studies

Even if there is convincing evidence showing that oxidative stress is directly involved in the pathophysiology of atherothrombosis in APS, very few studies have evaluated the potential effect of antioxidant supplementation in these patients.

One therapeutic approach could be represented by omega-3 polyunsaturated fatty acid (n-3 PUFA) supplementation, which has been shown to improve endothelial function in several diseases and could have a role also in APS. In a pilot study in 1993, 22 patients with persistent APS associated with recurrent miscarriage were treated with fish oil, equivalent to 5.1 g eicosapentaenoic acid (EPA) and docosahexaenoic acid (DHA) at a ratio of 1.5 EPA to DHA. The results showed that fish oil prevents recurrent miscarriage in persistent APS [59]. Different results were revealed by Carta et al. Patients with at least two consecutive spontaneous abortions and positive antiphospholipid antibodies on two occasions were assigned to treatment with low-dose aspirin or fish oil derivatives. Results showed that the treatment of women with recurrent pregnancy loss associated with APS syndrome with fish oil derivates or low-dose aspirin did not lead to significant differences with respect to pregnancy outcome and complications [60].

More recently, a clinical trial involving 22 adult women with primary APS randomized to receive placebo or n-3 PUFA (capsule, 1.8 g of EPA and 1.3 g of DHA). After 16 weeks of supplementation, the ω-3 group showed significant increases in endothelial function estimated by reactive hyperaemia index (RHI) when compared with placebo. In addition, the ω-3 group showed decreased circulating levels of interleukin-10 (−4 vs. +45%) and tumour necrosis factor (−13 vs. +0.3%) and a tendency toward a lower intercellular adhesion molecule-1 response (+3 vs. +48%) after treatment when compared with placebo. Conversely, no changes were observed for E-selectin, vascular adhesion molecule 1, and fibrinogen levels [61].

The effect of combined antioxidant treatment with Vitamin E and Vitamin C was investigated by Ferro et al. [41]. Eleven APS-positive patients were randomly supplemented either with or without antioxidants (vitamin E at 900 IU/day and vitamin C at 2000 mg/day) for 6 weeks. APL-positive patients showed increased oxidative stress that induced an overexpression of monocyte tissue factor (TF), contributing to activate the clotting system. Results showed that patients who received antioxidant supplementation had a significant reduction in isoprostanes and monocyte TF antigen and activity [41].

Stopa et al. evaluated the effect of flavonoid quercetins in a cohort of patients with persistently elevated antiphospholipid antibodies. Quercetin-3-rutinoside is a small, potent inhibitor of protein disulphide isomerase (PDI) that plays a critical role in thrombus formation. Oral administration of 1.000 mg isoquercetin decreased by 64% platelet-dependent thrombin generation in the antiphospholipid antibody cohort. Moreover, isoquercetin ingestion resulted in a decrease in the generation of platelet factor Va [62].

Finally, in a prospective, randomized, crossover, placebo-controlled trial, Perez-Sanchez et al. evaluated the short-term effects of in vivo ubiquinol, the reduced coenzyme Q_10_ [Q_red_], supplementation on biomarkers related to inflammation and thrombosis in APS. Thirty-six patients with APS were randomized to receive Q_red_ (200 mg/d) or placebo for 1 month. Results showed that Q_red_ (1) improved endothelial function and decreased the expression of prothrombotic and proinflammatory mediators by monocyte, (2) inhibited phosphorylation of thrombosis-related protein kinases, and (3) decreased peroxides and percentage of monocytes with depolarized mitochondria. Moreover, Q_red_ significantly reduces the percentage of neutrophil extracellular traps released by activated neutrophils, and in monocytes it downregulates peroxides production, intracellular elastase, and myeloperoxidase expression [63].

### 4.2. Experimental and In Vitro Studies

Murine experimental models of antiphospholipid syndrome (eAPLS) were used to test the effect of Omega-3 fatty acids and curcumin on neurologic severity. BALB/c mice immunized with beta-2-glycoprotein I received omega-3 fatty acids (0.5 g/kg), and curcumin (200 mg/kg) for 3 months in addition to treatment with enoxaparin (1 mg/kg). The enoxaparin and omega-3 fatty acids combination was correlated with a reduction in mortality, demonstrating an interesting therapeutic approach using omega-3 in eAPLS [64]. Another study evaluated the effect of 6-gingerol, one of the main functional compounds in the extract of ginger. The administration of antiphospholipid antibodies to mice increased thrombus length and weight, which returned to control levels upon administration of 6-gingerol (20 mg/kg, three times per week). The mechanism hypostatized is dependent on NETs formation. Indeed, NETs contribute to APS pathophysiology as amplifiers of inflammation and thrombosis. Indeed, the aPL administration increased serum NET levels, which returned to baseline when mice were treated with 6-gingerol [65].

Other data are provided by in vitro experiments. Ferro et al. evaluated the effect of Vitamin E on TF and oxidative stress in human healthy monocytes. When cells were treated with polyclonal anti-b2GP1 antibodies, a dose-dependent increase in oxidative stress, as indicated by the increase in superoxide anion and TF Ag and activity production compared to monocytes stimulated with IgG of normal healthy subjects, was observed. The pretreatment with vitamin E concentrations (50, 100 µM) dose-dependently reduced superoxide anion and TF Ag and activity production [41].

Wei et al. evaluated the effect of hyperoside, which is a flavonoid glycoside compound mainly found in medicinal herbs, displaying antioxidative, anticancer, and anti-inflammatory properties in many molecular pathways. Human umbilical vein endothelial cells (HUVECs) were treated with anticardiolipin antibody (aCL) to induce a vascular endothelial injury. When HUVECs were pretreated with hyperoside (10, 20, 50 mM) for 24 h, the secretion of proinflammatory cytokines, such as IL-1b and IL-8, and endothelial adhesion cytokines such as TF, ICAM1, and VCAM1, was significantly reduced. Mechanistically, hyperoside activated autophagy and suppressed the mTOR/S6K and TLR4/Myd88/NF-k B signalling transduction pathways [66].

Perez-Sanchez et al. evaluated the effect CoQ10 on mitochondrial dysfunction. The preincubation of human healthy monocytes with CoQ10, followed by treatment with IgG-APS, significantly decreased oxidative stress and the percentage of cells with altered mitochondrial membrane potential, suggesting a positive effect on alterations in mitochondrial dynamics and metabolism. Moreover, CoQ10 reduced the expression of the thrombotic and proinflammatory markers such as TF, vascular endothelial growth factor (VEGF) and its receptor Flt1, which were increased after the treatment with IgG-APS [58].

Finally, Wang et al. tested the effect of Epigallocatechin-3-gallate (EGCG), which is the major polyphenolic component of green tea, on blocking the effects of the anti-β2 glycoprotein I (GPI)/β2GPI complex. This complex activates endothelial cells and monocytes promoting TF activity, increasing the risk of thrombosis, and enhancing the expression and secretion of proinflammatory cytokines. Human acute monocytic leukaemia cell line (THP-1) treated with anti-β2 glycoprotein I (GPI)/β2GPI complex displayed increased expression of TF and tumour necrosis factor-α (TNF-α). When the cells were pretreated with EGCG (0–50 μg/mL) before the stimulation with the anti-β2GPI/β2GPI complex, TF expression and activity were significantly reduced [67].

## 5. Conclusions and Future Perspective

Oxidative stress is directly involved in the pathogenesis of atherothrombosis in APS patients. Several mechanisms have been proposed and have highlighted the role of aCL as a key promoter of oxidative stress and mitochondrial dysfunction. Oxidative stress, in turn, favours endothelial dysfunction, mainly associated with the alteration of NO metabolism, and stimulates a prothrombotic and proinflammatory status in APS patients.

Since the role of oxidative stress is well established, oxidative stress biomarkers should be used for thrombotic risk assessment in these patients and to plan antioxidant therapy for the reduction of thrombotic risk. In vitro studies are consistent in supporting a beneficial effect of treatment with antioxidants in reducing biomarkers of thrombosis. However, to date, there are little data on the administration of antioxidants in these patients, with no conclusive results. Therefore, further study with a more adequate methodology must be performed to assess the validity of antioxidant supplementation in patients with APS.

## Figures and Tables

**Table 1:** Clinical, experimental, and in vitro studies describing changes of biomarkers of oxidative stress in APS patients.

HUMAN STUDIES			
Author/(Year)/[Reference]	Study Type (Setting)	Markers of Oxidative Stress	Main Results vs. Controls
Lambert et al., (2000) [19]	n = 56 APS patients n = 71 HS	PON1 MDA-LDL	↓ PON1 ↑ MDA-LDL
Delgado Alves et al., (2002) [40]	Cross-sectional study n = 32 SLE n = 36 with PAPS n = 20 controls	HDL cholesterol PON activity TAC	↓ HDL ↑ anti-HDL antibodies ↓ PON activity ↓ TAC
Ferro et al., (2003) [41]	n = 13 APL patients n = 11 negative APL patients	Isoprostane	↑ 8-isoprostane
Matsuura et al., (2006) [42]	n = 93 APS patients n = 161 HS	oxLDL/beta2GPI	↑ oxLDL/beta2GPI
Sciascia et al., (2012) [43]	n = 45 APS patients n = 75 HS	Isoprostanes	↑ 8-isoprostane ↑ Prostaglandin E2 (PGE)
Perez-Sanchez et al., (2015) [44]	n = 126 APS patients n = 61 HS	TAC MnSOD Catalase GPx	↓ TAC ↑ MnSOD ↑ Catalase ↓ GPx
Stanisavljevic et al., (2016) [45]	Cross-sectional case–control n = 140 APS patients n = 40 HS	LOOH AOPP tSHG PON1	↔ LOOH ↑ AOPP ↓ tSHG ↓ PON1
Lai et al., (2015) [46]	n = 12 APS patients n = 54 HS	mitochondrial mass O_2_^−^ production mTOR and FoxP3 expression	↑ mitochondrial mass ↑ O_2_^−^ production ↔ mTOR expression ↓ FoxP3 expression
Ibrahim (2017) [47]	n = 75 APS patients n = 120 HS	polymorphisms of the PON1	↔ PON1 polymorphisms
Nojima et al., (2020) [48]	n = 58 APS patients n = 312 HS	OSI	↑ OSI
**EXPERIMENTAL STUDIES**			
Delgado Alves et al., (2005) [49]	mice with SCID+ aCL and anti-aβ2-GPI monoclonal antibodies	PON activity TAC	↓ PON activity ↓ TAC
Benhamou et al., (2015) [50]	APS mice	gp91phox mRNA GSH/GSSH ratio	↑ gp91phox mRNA ↑ left ventricular GSH/GSSH
Ding et al., (2015) [51]	APS mice wild-type mice	p47phox	↑ p47phox mRNA ↑ p47phox phosphorylation
**IN VITRO STUDIES**			
Ferro et al., (2003) [41]	Human monocytes from (HS) and anti-β_2_GP_1_ antibodies (50, 100, 200 µg/mL)	O_2_^−^ production	↑ O_2_^−^ production
Simoncini et al., (2005) [52]	HUVEC and IgG (IgG-APS) from 12 APS patients	ROS production	↑ ROS production MAP kinases pathway: ↑ p38 phosphorylation ↑ ATF-2

Abbreviations: healthy subjects (HS); Human umbilical vein endothelial cells (HUVEC); Mitogen-activated protein (MAP) kinases; activating transcription factor-2 (ATF-2); Oxidized low-density lipoprotein (oxLDL); b2-glycoprotein I (2GPI); prostaglandin E2 (PGE); Manganese-SOD, total antioxidant capacity (TAC); manganese-superoxide dismutase (MnSOD); glutathione peroxidase (GPx); lipid hydroperoxydes (LOOH); advanced oxidation protein products (AOPP); total sulfhydryl groups (tSHG); paraoxonase 1 activity (PON1); oxidation stress index (OSI); severe combined immunodeficiency (SCID); anticardiolipin (aCL); Total antioxidant capacity (TAC); ↑ increase; ↓ decrease; ↔ no changes.

**Table 2:** Main characteristics and main results of supplementation studies with antioxidants in patients with APS, experimental model of APS and in vitro studies.

INTERVENTION STUDIES			
HUMAN STUDIES			
Author/(Year)/[Reference]	Study Type (Setting)	Type of Intervention/Doses	Main Results
Rossi et al., (1993) [59]	Patients with PAPS associated with recurrent miscarriage n = 22	EPA and DHA (5.1 g)	Fish oil prevents recurrent miscarriage in persistent APS
Carta et al., (2005) [60]	A prospective study Patients with positive antiphospholipid antibodies n = 30	Fish oil derivates vs. low dose aspirin	No significant differences in adverse pregnancy outcome after fish oil derivates.
Felau et al., (2018) [61]	Randomized double-blind placebo-controlled trial Women with primary APS n = 22	EPA (1.8 g) and DHA (1.3 g) 16 weeks	↑ endothelial function ↓ circulating levels of interleukin-10 and TNF ↔ E- selectin, vascular adhesion molecule-1, and fibrinogen levels
Ferro et al., (2003) [41]	Randomized clinical trial APL positive patients n = 11	Vitamin E (900 IU day) Vitamin C (2000 mg day) 6 weeks	↓ Isoprostanes ↓ Monocyte TF antigen
Stopa et al., (2017) [62]	Clinical trial Patients with persistently elevated anti-phospholipid antibodies n = 6	Isoquercetin (1000 mg) 4 h	↓ thrombin generation (decrease of 63.6%) ↓ platelet factor Va generation
Perez-Sanchez et al., (2017) [63]	Prospective, randomized, crossover, placebo-controlled trial n = 36	Q_red_ (200 mg/d) vs. placebo 1 month	↑ endothelial function ↓ monocyte expression of prothrombotic and proinflammatory mediators ↓ peroxides
**EXPERIMENTAL STUDIES**			
Maalouly et al., (2017) [64]	Murine experimental models of antiphospholipid syndrome: BALB/c mice immunized with beta-2-glycoprotein I	Omega-3 fatty acids (0.5 g/kg) curcumin (200 mg/kg) 3 months in addition to the treatment with enoxaparin (1 mg/kg)	↓ mortality
Ramadan et al., (2021) [65]	Murine experimental models of antiphospholipid syndrome: esiquimod-induced (R848-induced) lupus	6-gingerol (20 mg/kg intraperitoneal injection) 3 times per week	↓ NETs release ↓ Anti-dsDNA, anti-β2GPI, and total IgG ↓ thrombus length and weight
**IN VITRO STUDIES**			
**Author/(year)/[reference]**	**Types of cells**	**Type of antioxidant’s treatment**	**Main results**
Ferro et al., (2003) [41]	Human healthy monocytes treated with polyclonal anti-b2GP1 antibodies	Vitamin E concentrations (50, 100 μM)	↓ superoxide anion ↓ TF Ag and activity
Wei et al., (2020) [66]	HUVECs treated with anticardiolipin antibody (aCL)	Hyperoside (10, 20, 50 mM)	↓ IL-1b, IL-8, TF, ICAM1, and VCAM1 ↑ autophagy ↓ mTOR/S6K ↓ TLR4/Myd88/NF-kB signalling transduction pathways
Perez-Sanchez et al., (2017) [63]	Human healthy monocytes treated with IgG-APS	CoQ10	↓ oxidative stress ↓ TF ↓ VEGF ↓ Flt1 receptor
Wang et al., (2014) [67]	Human acute monocytic leukaemia cell line treated with anti-β2 glycoprotein I (GPI)/β2GPI complex	Epigallocatechin-3-gallate (0–50 μg/mL)	↓ TF expression ↓ TF activity

Legend: anticardiolipin (aCL); β2-glycoprotein I (2GPI); docosahexaenoic acid (DHA); eicosapentaenoic acid (EPA); fms-related tyrosine kinase 1 (FLT1); glutathione peroxidase (GPx); human umbilical vein endothelial cells (HUVEC); intercellular adhesion molecule 1 (ICAM1); myeloid differentiation factor 88 (Myd88); mechanistic target of rapamycin (mTOR); nuclear factor kappa-light-chain enhancer of activated B cells (NF kB) primary antiphospholipid syndrome (PAPS); paraoxonase 1 activity (PON1); severe combined immunodeficiency (SCID); tissue factor (TF); Toll-like receptor 4 (TLR4); tumour necrosis factor-α (TNF-α); vascular cell adhesion molecule 1 (VCAM1); vascular endothelial growth factor (VEGF); ↑ increase; ↓ decrease; ↔ no changes.
